# A systematic review of theories, models and frameworks used for youth engagement in health research

**DOI:** 10.1111/hex.13975

**Published:** 2024-01-30

**Authors:** Sherald Sanchez, Rachel Thorburn, Marika Rea, Pamela Kaufman, Robert Schwartz, Peter Selby, Michael Chaiton

**Affiliations:** ^1^ Institute of Medical Science, Temerty Faculty of Medicine University of Toronto Toronto Ontario Canada; ^2^ Ontario Tobacco Research Unit, Dalla Lana School of Public Health University of Toronto Toronto Ontario Canada; ^3^ Department of Applied Psychology and Human Development University of Toronto Toronto Ontario Canada; ^4^ Centre for Addiction and Mental Health Institute for Mental Health Policy Research Toronto Ontario Canada; ^5^ Centre for Criminology and Sociolegal Studies University of Toronto Toronto Ontario Canada; ^6^ Department of Family and Community Medicine University of Toronto Toronto Ontario Canada

**Keywords:** patient engagement, theory, youth engagement, youth involvement in research

## Abstract

**Background:**

Youth engagement in research, wherein youth are involved in the research beyond mere participation as human subjects, is growing and becoming more popular as an approach to research. However, systematic and deliberate theory‐building has been limited. We conducted a systematic review to identify and synthesize theories, models and frameworks that have been applied in the engagement of youth in health research, including mental health.

**Methods:**

Six academic databases (MEDLINE, PsycINFO, Embase, PubMed, Scopus, CINAHL) and the grey literature were searched for relevant studies. Citation tracking was conducted through ancestry and descendancy searches. The final search was completed on 7 February 2023. Findings were summarized in a narrative synthesis informed by principles of hermeneutic analysis and interpretation. Reporting of results is in accordance with the PRISMA (Preferred Reporting Items for Systematic reviews and Meta‐Analyses) 2020 Statement.

**Results:**

Of the 1156 records identified, 16 papers were included, from which we extracted named theories (*n* = 6), implicit theories (*n* = 5) and models and frameworks (*n* = 20) used for youth engagement in health research. We identified theories that were explicitly stated and surfaced theories that were more implicitly suggested. Models and frameworks were organized into four categories based on their principal features: power‐focused (*n* = 8), process‐focused (*n* = 7), impact‐focused (*n* = 3) and equity‐focused (*n* = 2). Few frameworks (*n* = 5) were empirically tested in health‐related research.

**Conclusions:**

The state of theoretical development in youth engagement in research is still evolving. In this systematic review, we identified theories, models and frameworks used for youth engagement in health research. Findings from this systematic review offer a range of resources to those who seek to develop and strengthen youth engagement in their own research.

**Patient or Public Contribution:**

Youth engaged as patients in the research were not involved in planning or conducting the systematic review. However, youth researchers in their early to mid‐20s led the planning, implementation and interpretation of the review. As part of subsequent work, we formed a youth advisory board to develop a youth‐led knowledge mobilization intended for an audience of youth with lived experience of being engaged as patients in research.

## 1 INTRODUCTION

Patient engagement in research refers to a wide range of practices and approaches for involving patients in research beyond mere participation as a human subject. The term *‘patient’* extends to individuals with lived experience of a health condition or of navigating the health system.^1^ When involving youth populations, it is often referred to as youth engagement in research.^2^, ^3^, ^4^ Before gaining popularity among health researchers over the last two decades, youth engagement has been a focus in civic engagement studies and political participation research for at least 40 years.^5^ Its origins can be traced back to social movements and youth organizing, wherein young people are seen as drivers of social change.^6^ The core sentiment is embodied in the popular slogan ‘Nothing about us without us’.^7^, ^8^

In health sciences research, youth engagement is operationalized within programmes such as the Patient and Public Involvement (PPI) in Research in the United Kingdom,^9^ the Patient‐Centred Outcomes Research Institute in the United States^10^ and the Strategy for Patient‐Oriented Research (SPOR) in Canada.^11^ Engaging youth voices in research is a stated priority for funding institutions,^9^, ^10^, ^11^ scientific journals,^12^, ^13^, ^14^ hospitals,^2^, ^15^, ^16^, ^17^, ^18^ research decision‐makers^17^, ^19^, ^20^, ^21^ and government and nongovernmental organizations.^22^, ^23^, ^24^ Topics of studies in which youth were engaged include health promotion, particularly in tobacco and other drug use,^25^, ^26^, ^27^, ^28^, ^29^ digital health interventions,^30^, ^31^, ^32^, ^33^, ^34^, ^35^ sexual health,^36^ human immunodeficieny virus (HIV) research implementation,^37^, ^38^ early psychosis^39^, ^40^ and harm reduction.^29^, ^41^, ^42^

In the existing literature, the involvement of youth in health sciences research has been linked to far‐reaching benefits,^43^, ^44^, ^45^, ^46^ generally framed in terms of benefits to the youth themselves (youth empowerment, positive youth development),^30^, ^47^, ^48^, ^49^, ^50^ impact on the conduct and outcomes of research (methodological rigour, external validity)^11^, ^51^ and the advancement of policy objectives with respect to involving the public in research (EDI principles).^52^, ^53^ One of the most commonly stated benefits of engaging youth in research is that they provide unique perspectives that help enhance research questions and priorities.^1^ Others have argued that youth engagement in research will result in increased translation, dissemination and uptake of research findings.^54^, ^55^ There are also those who argue that youth engagement will lead to positive outcomes in youth development, political democracy and health equity.^30^, ^47^, ^48^, ^49^, ^50^ Motivations for youth engagement also include epistemic justice, which examines how knowledge is formed and emphasizes the importance of including diverse perspectives and experiences in the production of knowledge.^56^ Yet, the mechanisms through which these benefits are achieved have rarely been articulated or tested, highlighting the lack of evaluation studies as one of the most critical gaps in the literature.^57^, ^58^, ^59^

Despite the rise in popularity and continued growth of youth engagement in health research, systematic and deliberate theory‐building has been limited.^43^, ^60^, ^61^, ^62^ Theoretical development is crucial to advance this field of study towards an effective ‘science of engagement’, which can improve transparency, reliability and rigour in the research process. It is important to understand the different theories about youth research engagement in the literature. Therefore, the primary purpose of this systematic review was to identify and synthesize theories, models and frameworks applied in the engagement of youth in health research, including mental health.

Given the shortage of named theories in our initial searches, we expanded our scope to include models and frameworks as relevant forms of theory involving systematic thinking around youth engagement in research. Further, while the explicit use of theory was scarce in the literature, there was an abundance of beliefs, values and motives. These were included in our analysis as implicit theories, given the ways in which they influence youth engagement in research, including how evidence is collected, interpreted and applied.^63^ This deserves attention because when theories are implicit, ‘their power to clarify or to confuse, and to reveal or obscure new insights, can work unnoticed’.^63^ In general, these theories, models and frameworks can be referred to as theoretical approaches. To our knowledge, this is the first systematic review of theories, models and frameworks used for youth engagement in research.

## 2 METHODS

We followed the PRISMA (Preferred Reporting Items for Systematic reviews and Meta‐Analyses) 2020 Updated Guideline for Reporting Systematic Reviews (Material S2).^64^ The review protocol was registered before the conduct of the review (PROSPERO 2021 CRD42021234059).^65^ There were no significant deviations from the review protocol.

### 2.1 Data sources

We searched six academic databases (MEDLINE, PsycINFO, Embase, PubMed, Scopus and CINAHL) for relevant studies using a combination of search terms and subject headings to capture youth engagement in research. In accordance with best practices on updating systematic review searches,^66^ we reran our searches periodically between December 2021 and February 2023 to check for relevant literature that should be noted. The most recent search was completed on 7 February 2023.

We hand‐searched academic journals focused on topics directly relevant to the review question, specifically Health Expectations, BMC Health Services Research, BMC Research Involvement and Engagement, Children and Youth Services Review and Journal of Adolescent Health. We also hand‐searched relevant grey literature archives in Canada, including the websites for the Ontario Centre of Excellence for Child and Youth Mental Health, the Centre for Excellence for Youth Engagement, the Ontario Tobacco Research Unit and the McCain Centre's Youth Engagement Initiative. We also checked the reference lists of the papers that met our eligibility criteria. Then, we performed citation tracking through both ancestry and descendency searches by looking up each paper on Google Scholar to track down earlier work on which the papers were based (‘ancestor’ studies) and to identify more recent studies that cited the included papers (‘descendant’ studies).

### 2.2 Eligibility criteria

Three reviewers (S. S., M. R., M. C.) screened titles and abstracts independently. Disagreements were resolved through discussions among the three reviewers. After establishing a substantive level of agreement in the application of the eligibility criteria, two reviewers (S. S., M. R.) conducted full‐text screening independently. Disagreements were resolved through thorough in‐depth discussions between the two reviewers, and with the senior team member (M. C.) whenever needed.

Studies were included if they contained an explicit reference to a named theory, model or framework used for youth engagement in health research, including mental health. We applied the World Health Organization (WHO) definition of youth as individuals in the 15–24 age group.^67^ With respect to the concept of engagement, the terminology used in the literature is vast and diverse^43^; thus, we followed the SPOR definition of patient engagement in research as the meaningful and active collaboration between patients and researchers in research governance, decision‐making and the conduct of research itself from agenda‐setting to knowledge translation.^1^ There were no date or geographical restrictions. Relevant articles in English were included.

Studies were excluded if they did not involve youth in a health‐related research context and if there was no explicit reference to a youth engagement theory, model or framework. Studies focused on youth engagement in direct health services instead of health research were also excluded.

### 2.3 Data extraction

Two reviewers (S. S., M. R.) performed data extraction independently. Data were extracted from the manuscript, as well as the model or framework for youth engagement in research depicted in the study. Disagreements were resolved through discussions among the two reviewers (S. S., M. R.) and the senior member of the research team (M. C.).

We used a data extraction form developed a priori to identify key aspects of each study, namely, study characteristics (title, author, publication date, setting), funding, rationale for the development of the model or framework, definition of engagement provided by the authors, theoretical basis (if any) and evidence of framework use in empirical or other theoretical work. As an additional step, we also extracted data from the visual representations of the models to complement the data extracted from the main text and to make explicit certain elements that were absent or only implied in the main body of the text. One area where this proved to be a crucial step was in frameworks that depicted the process of engagement using ladder‐ or continuum‐type visuals, leading us to interrogate the direction of the arrows (unidirectional or bidirectional) and the meaning placed in the levels of engagement (hierarchical or otherwise).

Given the heterogeneity of the studies and the purpose of the systematic review, no quality assessment was conducted. The models and frameworks included in this systematic review can be found in Material S1.

### 2.4 Data synthesis

Our narrative synthesis was produced using hermeneutic analysis and interpretation, which involved an iterative process of searching for relevant literature, analytical reading, mapping and classifying, critical assessment and problem formulation.^60^, ^68^, ^69^, ^70^, ^71^ As we aimed to identify and synthesize different types of theoretical approaches used for youth engagement in health research, our findings were broken down into three main sections: explicit theories, implicit theories and models and frameworks. First, we described named theories used for youth engagement in health research as identified from the included studies. Second, we grouped the different types of models and frameworks that we identified in the literature based on their key characteristics. We followed Greenhalgh et al.'s approach of categorizing frameworks used for PPI among adult populations, and we developed a classification system for youth engagement in research.^60^ Lastly, we identified and summarized implicit theories (i.e., beliefs, motives, values) applied in youth research engagement into five categories, namely, substantive, instrumental, normative, political and developmental. We adopted the first four categories from the work Oliver et al. in PPI,^57^ and then added ‘developmental’ as a fifth category specific to youth involved in research.

## 3 RESULTS

**Figure 1:**
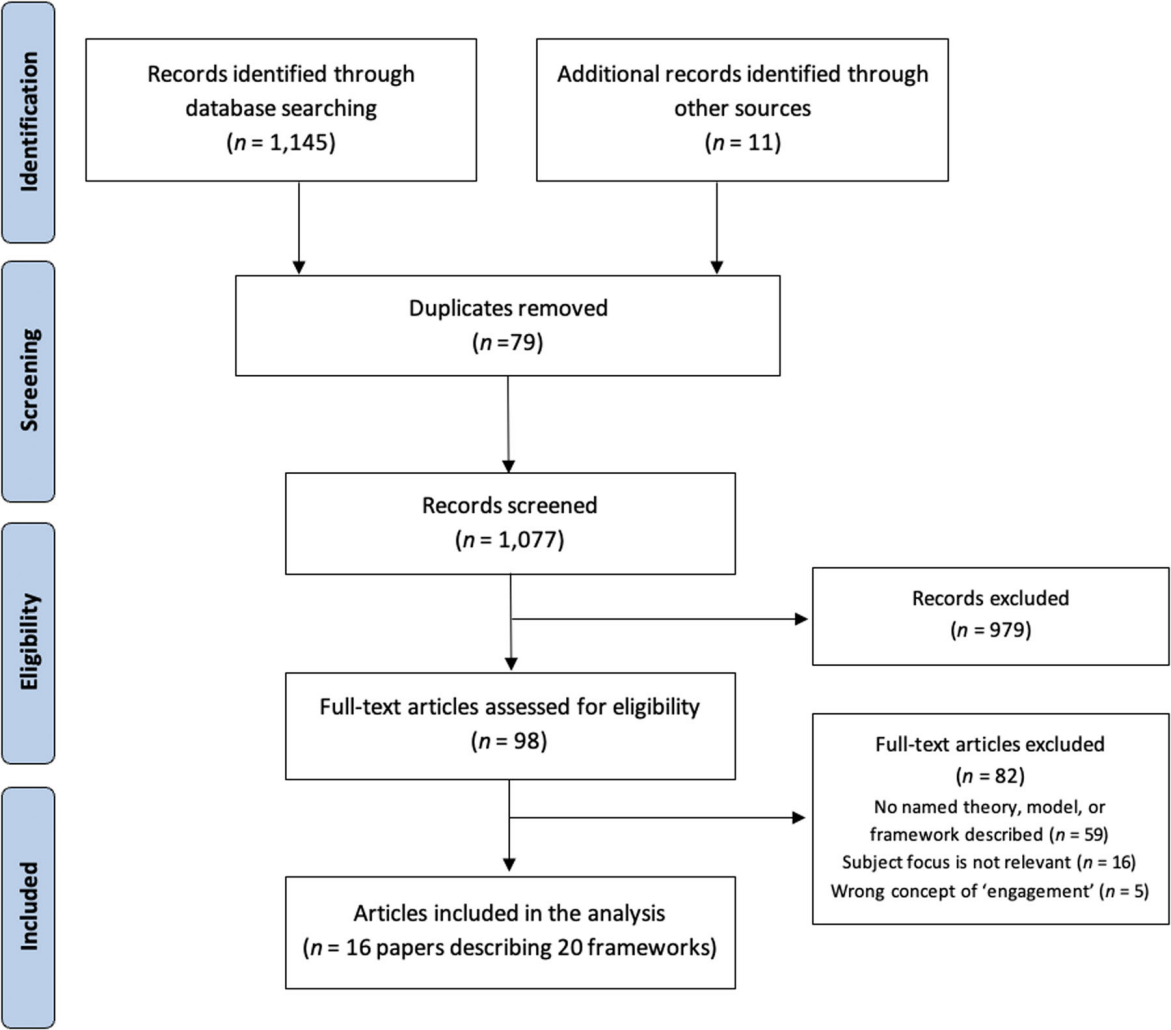
PRISMA (Preferred Reporting Items for Systematic reviews and Meta‐Analyses) flow diagram of the study selection process.

The PRISMA flow diagram describing the study selection process is shown in Figure 1. Through the search strategy, 1145 records were identified; through ancestry and snowball searches, 11 additional papers were found (total *n* = 1156). After removal of duplicates, 1077 abstracts were screened for eligibility. Of these, 98 papers met the inclusion criteria and were retrieved for full‐text screening. After applying the exclusion criteria, 16 papers describing 20 models and frameworks from five countries were included in the final data set.

Wherever authors specifically used a term (i.e., model or framework) to describe their work, we applied the term that they used. However, it was more common that the terms model and framework were used interchangeably; thus, they are used interchangeably throughout the text. Key characteristics of the included studies are described in Table 1.

**Table 1:** Key characteristics of the included studies.

Study characteristics	Number of studies (total *n* = 16)
Country	
United States	7
Canada	4
United Kingdom	2
Australia	2
Denmark	1
Publication type	
Essay	1
Training manual	1
Book chapter	1
Academic paper describing framework development	4
Academic paper with empirical examples	7
Review article	2
Discipline	
Psychology	4
Sociology	3
Health education	2
Public health	5
Interdisciplinary, youth‐focused	2
Research paradigm	
Participatory research or participatory action research	11
Not specified	5
Theoretical or conceptual basis	
Freire's critical pedagogy or pedagogy of the oppressed	3
Bandura's theory of self‐efficacy	1
Bronfenbrenner's ecological systems theory	1
Vygotsky's sociocultural theory of learning	1
Democratic theory	1
Empowerment theory	1
Not specified	8
Empirical testing in the included study	
Yes	7
No	9
Target audience	
Professionals (e.g., researchers, practitioners of youth engagement)	3
Both professionals and youth	4
Not specified	9
Framework development	
Developed with youth	2
Not specified	14

### 3.1 Explicit theories

Overall, explicit theory use was not common in the literature. Compared to studies that referred to a named theory, the application of conceptual models and frameworks was more prevalent. As shown in Table 1, six named theories were identified.

With three studies^27^, ^34^, ^44^ referring to it, Freire's critical pedagogy or ‘pedagogy of the oppressed’^72^, ^73^ was the only theory that appeared more than once. The theory's central premise is the liberation of oppressed and exploited communities from oppressive elements in society by their own hands and through their own actions. This includes liberation from economic, social and political forces, including liberation from unequal forms of knowledge production and distribution. One of the authors citing Freire suggested that this theory is closely connected with youth empowerment, specifically that which is derived from individual agency and collective participation combined in research where both youth and adults are involved.^44^

Each of the remaining five named theories appeared once. Bandura's social theory of self‐efficacy^74^, ^75^ was used to understand the impact of youth‐led research, evaluation and knowledge application in adolescent health issues.^49^ The authors of the included paper claimed that offering youth the opportunity to understand their own experiences enabled youth agency and informed action towards effective social change.^49^

Bronfenbrenner's ecological systems theory^76^ emphasized the role of contextual variability in providing insight into human development. Based on this, an ‘ecologically valid’ study of an individual involves the study of their environment and the systems in which they belong (e.g., social class, ethnicity, culture). In the context of youth engagement in research, the theory was applied to inform criticisms of the field at that time, wherein ‘much of the existing research on youth participation has been piecemeal, in which only small segments of the involvement process have been examined in relative isolation’.^47^

According to Vygotsky's sociocultural theory of learning,^77^ feeling (affect) is equally important as thinking (intellect) in mental and psychological development. Given this, youth engagement in research and programme development is essential in promoting learning as ‘a way of being in the social world, not coming to know about it’.^78^ In the included paper, the theory was applied to youth participation in health education.^78^

Democratic theory was generally described in terms of democratic principles by which citizens have the means to change or to consider changing their government.^48^ Hart applied this theory in a critique of ‘traditional educational practices’, arguing that young people in schools were being offered ‘a fixed set of beliefs’ as a form of ‘political indoctrination’.^48^ Hart claimed that genuine participation was an ‘antidote’ to this.^48^ Through genuine participation in projects, youth can be involved in problem solving for real‐world issues, developing critical thinking skills essential to their political self‐determination and the democratization of society.

Lastly, empowerment theory^79^, ^80^ was briefly described as a lens for assessing youth participatory approaches in terms of the degree to which they created empowering conditions for youth at the individual, organizational and community levels.^50^

### 3.2 Implicit theories

We identified implicit theories that explained different arguments, beliefs, motives and values with respect to youth engagement in health research. We organized them into the following five categories.

1. *Substantive*: Engagement is undertaken to enhance the quality of research, policy and practice. As Oliver et al.^57^ describes, ‘this may be through helping researchers and policy‐makers develop a more holistic understanding of a context, an issue and/or a solution, both epistemologically and ontologically’. Among the included studies, researchers made links between engaging youth in research and increasing the relevance of research questions,^46^, ^81^ health promotion and education^27^, ^34^, ^48^, ^50^, ^78^, ^82^, ^83^ and intervention development.^27^, ^34^, ^37^, ^50^ While the exact mechanisms by which research may be improved were not always clear, many agreed that engagement makes research more relevant by turning the focus on pertinent issues and by highlighting the importance of nonacademic perspectives in discovering ‘unknown unknowns’ and producing collaborative knowledge.^46^, ^57^, ^83^
2. *Instrumental*: These motivations were the most directly related to applied health services research and can be characterized by a strong interest in seeing research findings applied and used in tangible ways—for example, in behaviour change interventions^27^, ^37^ and health promotion programmes targeted towards young people.^34^, ^47^, ^78^, ^82^, ^83^ Engagement is focused on the context of implementation and driven by practice‐based questions and outcomes, such as how to foster patient trust towards a new intervention, or how to educate clinicians and nonacademics about emerging research. These questions and outcomes aimed to help push new research into implementation or understand how existing implementation could be improved or better received.
3. *Normative*: Engagement is seen as a human right with intrinsic value connected to democracy and the discourse on public–academic partnerships, focused on increasing transparency and accountability in the use of public funds and ensuring that research is carried on to serve public interests.^34^, ^44^, ^48^, ^84^, ^85^ The motivation for engagement went beyond recognizing youth as valuable resources for research and into an understanding that when youth are meaningfully engaged, ‘research evolves from a mere academic exercise into a social action process for achieving social change that can impact the root causes of health disparities’.^49^ These arguments can be traced back to Hart,^48^ who emphasized that ‘there are additional and more important benefits to a society beyond the short‐term one of making a programme or product more appropriate for the user. Unfortunately, these benefits have the kind of indirect, long‐term impact that cannot be easily measured quantitatively’.
4. *Political*: Engagement is political as evident in its focus on social justice and changing the beliefs and attitudes of different groups (often those holding power) to attain a particular end (often involving the disempowered).^34^, ^44^, ^49^, ^84^ For example, describing their experiences of engaging youth in HIV research, Asuquo et al.^37^ highlighted the value of engagement in changing attitudes and facilitating behaviour change towards reducing stigma. Drawing from social epidemiology, specifically the ecosocial theory of disease and distribution, one paper^50^ emphasized that engagement research ‘examines the extent to which actors, such as youth, are empowered to produce knowledge and develop interventions for addressing health inequities’. Generally, political arguments were grounded in historical perspectives of academia as an institution that privileges some forms of knowledge over others.^27^, ^34^, ^48^, ^83^, ^86^ Political arguments were less explicitly stated, albeit hinted to, by researchers.
5. *Developmental*: Engagement is associated with positive youth development to help young people achieve their full potential.^27^, ^47^, ^48^, ^82^, ^86^ Capacity building and positive youth development were the most frequently cited motivations for engaging youth in research. Specifically, engagement was thought to provide youth with affective relationships and diverse experiences, helping them develop social, emotional and cognitive competencies, which can also act as protective factors against health‐related issues, such as disease, violence and substance use.^46^, ^49^, ^82^, ^86^ Researchers frequently cited evidence suggesting that youth engaged in research are less depressed, have higher self‐esteem, are more physically active, obtain higher grades in school and show a greater commitment to their friends, families and communities.^27^, ^46^, ^48^, ^82^, ^86^

### 3.3 Models and frameworks

Models and frameworks were more common in the literature relative to named theories. We identified five applications of models and frameworks used for youth engagement in health research: (1) empirical testing by the groups that developed them,^37^, ^46^, ^49^, ^50^, ^83^ (2) informing subsequent work on specific categories of the framework,^48^, ^50^, ^83^, ^84^, ^85^ (3) strengthening endorsements and calls for increasing youth engagement in research,^27^, ^34^, ^44^, ^48^, ^49^, ^50^, ^81^, ^82^, ^83^, ^84^, ^85^ (4) supporting arguments in review and commentary articles^44^, ^48^, ^49^, ^50^, ^81^, ^83^, ^84^, ^85^, ^86^ and (5) developing tools for evaluating youth engagement in research.^44^, ^47^, ^78^ While the models and frameworks had overlapping characteristics, we grouped them into four classification types according to their primary focus. This classification system is summarized in Table 2.

**Table 2:** Classification of models and frameworks used for youth engagement in research.

Categories	Characteristics of frameworks	Observations
Power‐focused frameworks	Identifying, describing and explaining the allocation of power and control between youth and adult researchers in academic research	Generally concerned with who has control and leadership in a project involving partnership between youth and adult researchers; tend to ask political questions aimed at challenging prevailing structures of power in science and academia that privileges academic expertise over almost all other forms of expertise
Ladder of Children's Participation^48^		
Degrees of Participation^84^		
Pathways to Participation^85^		
TYPE Pyramid^44^		
YPAR People Powered Place‐Making^34^		
Authentic Youth Participation^82^		
Levels of Youth Participation^81^		
Measure of Youth Engagement^37^		
Process‐focused frameworks	Describing barriers, facilitators and other factors affecting youth engagement in research. Identifying recommendations, principles and methods for maintaining research integrity and maximizing the benefits of youth participation in the research process, specifically in the context of applied health research, whereby research‐based inquiry is informing an ‘action’ component, such as health promotion or intervention design	Process‐focused frameworks offer a detailed description of the different strategies, factors and components involved in youth engagement in research.
Youth Agency for Social Change Model^49^		
Model of Student Participation^78^		
YPAR Continuum^34^		
McCain Centre Model of Youth Engagement^46^		
P7 Model^86^		
Youth Engagement for Community Change^82^		
Making Youth Participation Genuine^81^		
Impact‐focused frameworks	Conceptualizing and understanding the links between engaging youth in research and the different short‐ and long‐term impacts associated with it	Aimed at understanding and evaluating the impact of youth engagement in research: exploring the links between engagement and positive youth health and development guided by a ‘what, who, where, when, and why’ framework
Youth Engagement Framework^83^		
EIPARS Model^27^		
Youth Engagement Framework by the CEYE^47^		
Equity‐focused frameworks	Identifying and proposing alternative approaches to conventional academic research methods that may be incompatible with or a hindrance to fulfilling the objectives and principles of youth engagement in research	Challenges the status quo in conventional academic research and considers alternative forms of knowledge production; different from process‐focused frameworks that devote resources to finding ways to work *within* the current system
YPAR 2.0 Model of Research Engagement^34^		
Youth Participation Approaches Decision Tree^50^		

Abbreviation: CEYE, Centre of Excellence for Youth Engagement; TYPE, Typology of Youth Participation and Empowerment; YPAR, youth participatory action research.

#### 3.3.1 Power‐focused

Five models and frameworks from the United States,^34^, ^37^, ^44^, ^48^, ^82^ two from the United Kingdom^84^, ^85^ and one from Australia^81^ (*n* = 8) were primarily concerned with identifying, describing and sometimes explaining who has control and leadership in a project involving partnership between youth and adult researchers. These models offered a visual interpretation of power dynamics between youth and adults in health research, with the most common type taking on a ladder^37^, ^48^, ^82^ or ladder‐like shape^81^, ^85^ (e.g., a unidirectional continuum, essentially a ladder turned sideways). The classical example of the ladder model is Hart's Ladder of Children's Participation,^48^ which was published in 1992 and based on Arnstein's Ladder of Citizen Participation first published in 1969.^87^ Hart's Ladder described total youth control—positioned at the top of the ladder—as the ideal representation of power. In contrast to the ladder model, the Typology of Youth Participation and Empowerment (TYPE) Pyramid conceptualized the distribution of power between youth and adults through a pyramid: with adult control on one slope and youth control on the other, the TYPE Pyramid presented shared control between youth and adults as the target, visually represented as the peak of the pyramid.^44^ All power‐focused frameworks clearly identified Hart's Ladder as their starting point.

The Degrees of Participation^84^ imagined different forms of engaging youth in research based on the degree of decision‐making power held by youth at various stages of the research cycle. The model took the form of a web, a conscious decision by Treseder to move away from prescriptive notions of youth engagement. Treseder argued that full youth control may be unsuitable in some cases, and it is more practical to identify five distinct yet equal forms of participation, namely, assigned but informed, consulted and informed, adult‐initiated (shared decisions with youth), youth‐initiated (shared decisions with adults) and youth‐initiated and ‐directed.^44^

#### 3.3.2 Process‐focused

Three models and frameworks from the United States,^34^, ^49^, ^82^ two from Australia,^81^, ^86^ one from Canada^46^ and one from Denmark^78^ (*n* = 7) focused on understanding how to engage youth in conducting research, describing relevant factors and identifying recommendations and principles for maintaining research integrity and maximizing the benefits of youth participation. The earliest process‐focused framework identified was the Youth Agency for Social Model published in 2006.^34^ This model was developed to guide seven projects in school‐based health centres focused on adolescent health issues. A second model called Making Youth Participation Genuine^81^ was a result of qualitative interviews with youth mental health researchers in Australia exploring individual and organizational factors that act as barriers and facilitators to youth engagement in mental health research.

The P7 Model^86^ illustrated the dynamic interaction between seven domains affecting youth participation in research or programme planning, namely, place, process, perspective, purpose, protection, positioning and power relations. The YPAR (youth participatory action research) Continuum^34^ described three YPAR approaches and corresponding technology‐based tools for engaging youth in research from ‘analog surveys’ in YPAR to ‘digital photography’ in electronic participatory action research, then to ‘real‐time crowd‐sourced mobile tools’ in YPAR People Powered Place‐Making. The Model of Student Participation ^78^ identified three points of differentiation between ‘token’ and ‘genuine’ youth participation in school‐based health promotion, namely, focus, outcomes and target of change. The model also focused on the process of collecting, analysing and contextualizing health data in ‘intentional, relational, and purposeful ways’ that foster genuine youth participation.^78^

#### 3.3.3 Impact‐focused

Three models and frameworks from Canada^27^, ^47^, ^83^ (total *n* = 3) focused on describing and conceptualizing the potential impacts of youth engagement in research. These explored the links between engagement and engagement outcomes guided by a ‘what, who, where, when, and why’ framework. Pancer et al.,^83^ as a response to the focus on individual outcomes at the time, investigated outcomes associated with youth engagement at the systems level. Self‐esteem was identified as an individual outcome.^83^ On the other hand, an example of a systems‐level outcome identified was a change in the organizational structure and priorities of funding agencies towards being more inclusive and representative with regard to health promotion among youth.^83^ The Youth Engagement Framework^83^ developed by Pancer and colleagues is the earliest impact‐focused framework included in this review, followed by the Youth Engagement Framework developed for the Centre of Excellence for Youth Engagement (CEYE), a youth‐focused organization based in Canada after which the model was named.^47^

Both frameworks described three core elements of engagement: (1) affective, involving emotional and subjective responses to participation; (2) cognitive, pertaining to knowledge and thoughts gained through participation; and (3) behavioural, referring to actions related to participations, such as attendance to an event.^47^ The author used these elements to distinguish between mere participation or what is referred to as ‘activity involvement’, whereby engagement involves activation only in the behavioural aspects without any psychological engagement. Rose‐Krasnor argued that impactful engagement requires the activation of all three multidimensional components.^47^

Lastly, the EIPARS Model^27^ depicted a six‐step approach to youth engagement: engage (connect with interested youth); identify (youth identify an issue); plan (youth develop a plan to address the issue); act (youth implement the plan); research, reflect and reward (youth evaluate their strategies and reflect on the outcomes); and sustain (youth identify strategies for sustainability). As an impact‐focused framework, the model also described three interrelated levels of project impact: the direct impact of the research project on youth participants; the project's impact on the local community comprising of family, peers, school and town; and the impact of the project on a population level.

#### 3.3.4 Equity‐focused

Two models and frameworks^34^, ^50^ from the United States were classified as equity‐focused frameworks. The Youth Participation Approaches Decision Tree^50^ explored different youth participatory approaches—such as YPAR, human‐centred design and youth advisory boards—and their impact on health equity. On the other hand, the YPAR 2.0 Model of Research Engagement^34^ focused on developing a model that positioned research as a tool to co‐produce knowledge with communities, rather than using research as a way to test a hypothesis predetermined by researchers.

The two frameworks shared several core components: (1) the premise that young people's perspectives and experiences are needed for a more holistic understanding of key issues affecting their health and well‐being, (2) emphasis on challenging neoliberal notions of ‘expert knowledge’ and recognizing ‘local knowledge’ that is youth‐centred and crowd‐sourced, (3) the conviction that participatory approaches support critical scientific inquiry that can improve the accuracy and rigour of evidence, rooted in a thorough understanding of the issues that youth are facing to define lasting solutions,^50^ (4) close attention to the social determinants of health and (5) roots in social justice and the centrality of structural issues and their greater impact on youth health and well‐being over individual behaviour change.

## 4 DISCUSSION

**Figure 2:**
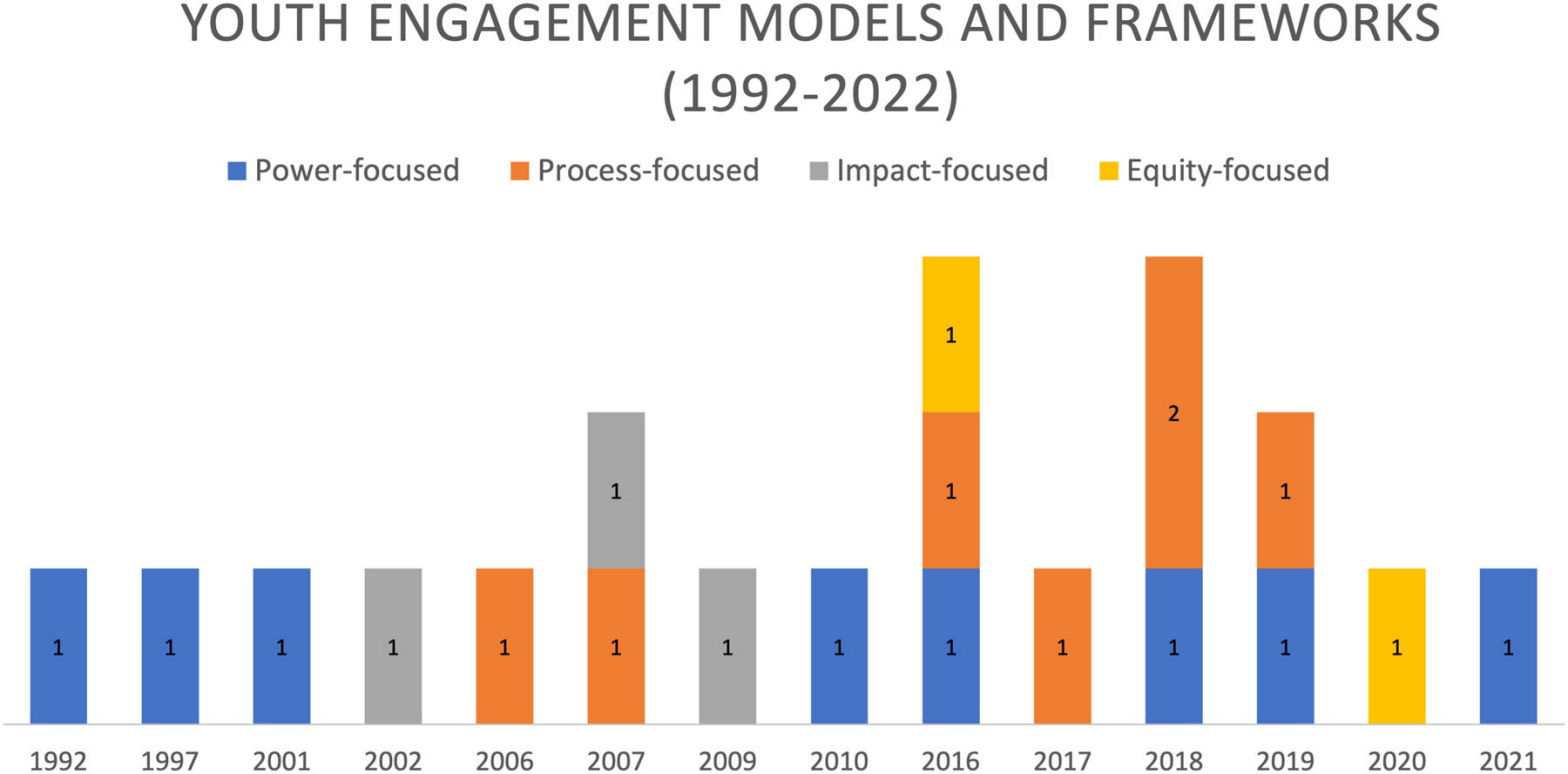
Youth engagement in research models and frameworks published annually.

To our knowledge, this is the most comprehensive and systematic summary of theoretical approaches used for youth engagement in health research. In discussing review findings, it is important to highlight the evolution of youth engagement frameworks since Hart's Ladder of Children's Participation,^48^ as observed in Figure 2. Three decades after Hart's Ladder of Children's Participation was published, it continues to be the most influential conceptualization of young people's engagement in research. However, there is growing recognition that power‐focused frameworks may be insufficient in advancing this area of research. Some have argued that power‐focused frameworks like Hart's Ladder may be useful ‘when considering the amount of influence desired in a study, but it obscures the goal of improving the research’.^88^ Hart himself recognized this and called for other scholars to use the ladder as a ‘jumping‐off point’ to develop future models capturing the complexities of engaging youth in research.^89^ Scholars of youth engagement have demonstrably taken up Hart's call over the course of three decades. Surely, this is an evolving field, and our knowledge base will continue to grow as more work is published.

Next to power‐focused models and frameworks, process‐focused frameworks are being developed at a fast pace. We identified process‐focused frameworks concerned with understanding more enduring factors affecting youth engagement in research, such as culture and institutions.^27^, ^49^, ^81^ Others devoted more attention to elements that are swiftly changing over time, such as technology, specifically the influence of new means of mass communication and information dissemination.^34^ We suspect that process‐focused frameworks will continue to grow.

Given the near‐unanimous recognition that the biggest gap in this area of research is the lack of evidence on impact gained through rigorous evaluation,^43^, ^57^, ^58^, ^61^, ^90^ the small number of impact‐focused models and frameworks is noteworthy. These frameworks emphasized the importance of systems‐level outcomes and highlighted that achieving these longer term outcomes will require innovative and critical thinking around youth engagement in research.^27^, ^47^, ^83^ This is consistent with review findings of empirical studies in PPI whereby short‐term, individual‐level outcomes were more represented in the literature.^43^, ^91^, ^92^

Of the four types of frameworks, equity‐focused frameworks represent the most recent development. This seems reflective of the ever‐increasing interest in equity, diversity and inclusion (EDI) within health research and health services.^50^, ^51^, ^93^ Applying an equity lens, findings from this review showed that framework development is concentrated in high‐income countries. If there is little consideration of the applicability of theoretical approaches to research conducted in low‐resource settings or other cultural contexts, then this is a major limitation in the field. A similar statement can be made for high‐resource settings where researchers should be involving young people from diverse socioeconomic and cultural backgrounds.

Perhaps, a concerning trend is the lack of youth engagement in the development of the theories, models and frameworks that are focused on how they should be involved in health research. In our tally, only two studies explicitly stated having involved youth in the development of their framework, while the rest did not specify whether youth were involved in their framework development process. This highlights two issues: first is the lack of youth engagement in theory and framework development and second is the inconsistencies in the reporting of youth engagement in research. As the field grows, it is important to assess whether the approaches that we have are appropriate and useful for a range of diverse youth and how well they have been considered throughout the development and implementation of these models and frameworks.

We observed that the use of theory was more common in the psychology discipline, particularly community psychology.^44^, ^50^, ^80^ Given the interdisciplinary nature of youth engagement in research, it is essential for health sciences researchers to map engagement to its epistemological roots, including Freire's pedagogy of the oppressed^72^ and community‐based participatory research.^94^ If we are not careful, patient engagement in research may turn into just another trend, losing its potential to effect lasting change with tangible benefits for all, especially marginalized populations.^95^

Overall, the state of theory‐building in youth engagement in research is still evolving, and more research is needed to refine and expand existing theories. Review findings demonstrated that there is little need to reinvent the wheel when it comes to theoretical approaches in this area of research. Rather, a more deliberate theory‐building is needed to help advance the field. Deliberate theory‐building can lead to more vibrant and critical discussions for the research community to interrogate its assumptions about youth engagement. Findings can aid the research community in the selection and application of theoretical approaches in engaging youth in research, as well as in the planning of new initiatives and funding criteria, monitoring and evaluation and reporting in journal articles.

### 4.1 Strengths and limitations

This work has strengths and limitations. This is the first systematic review to summarize the state of theoretical work in youth engagement in health research. This work resulted in comprehensive findings that highlighted the dynamics between what is explicitly stated and what is implied in the literature on youth engagement in health research. Our findings may also be relevant to other areas of research beyond health sciences and health services. With that said, our focus on theoretical approaches (though important for many reasons already stated) meant that we excluded empirical studies that did not explicitly refer to a theory, model or framework of youth engagement in research. Further research into the empirical body of knowledge is warranted, for example, this recent systematic review of youth engagement in mental health research.^4^ Hand searching of the grey literature was focused on Canadian sources; hence, it is likely that other international examples were missed. At the time that this study was conducted, the authors were based in Canada and were principally familiar with the local landscape of youth research engagement. In Canada, the dominant term is ‘youth engagement in research’. Finally, and most importantly, youth engaged as patients in the research were not involved in planning or conducting the systematic review. The next steps for this research will include a knowledge dissemination strategy led by youth, for a youth audience.

## 5 CONCLUSIONS

The state of theoretical development in youth engagement in research is still evolving. In this systematic review, we identified theories, models and frameworks used for youth engagement in health research. Findings from this systematic review offer a range of resources to those who seek to develop and strengthen youth engagement in their own research.

## AUTHOR CONTRIBUTIONS

**Sherald Sanchez**: Conceptualization; investigation; writing—original draft; methodology; visualization; writing—review and editing; formal analysis; project administration; data curation; supervision. **Rachel Thorburn**: Writing—original draft; writing—review and editing; formal analysis. **Marika Rea**: Writing—original draft; investigation; formal analysis. **Pamela Kaufman**: Writing—review and editing; validation. **Robert Schwartz**: Writing—review and editing; validation. **Peter Selby**: Writing—review and editing; validation. **Michael Chaiton**: Conceptualization; supervision; writing—review and editing; resources; investigation; methodology; validation.

## CONFLICT OF INTEREST STATEMENT

The authors declare no conflict of interest.

## POSITIONALITY STATEMENT

When this was written, the first, second and third authors were graduate students and youth researchers in their early to mid‐twenties. The first author's doctoral training is primarily in health sciences; the second author is trained in clinical psychology and education; and finally, the third author was a graduate student in criminology and sociolegal studies. The rest of the authors were mid‐ or late‐career academics in public health and medicine, particularly mental health and addictions, who provided valuable insight and expertise in the study as part of the first author's doctoral advisory committee. The authors have varying degrees of experience in the practice and study of youth engagement in health research. Coming from a multiplicity of disciplines and experience, the authors were brought together by a desire to comprehend the state of theoretical work on patient engagement in research focused on youth.

## Supporting information

Supporting information.

Supporting information.

## Data Availability

The authors confirm that all data generated or analysed during this study are included in this published article.
