# Supplementary material for: A systematic review of theories, models and frameworks used for youth engagement in health research
Source: Health Expect. 2024 Jan 30;27(1):e13975. doi: 10.1111/hex.13975 (PMC10825621; doi:10.1111/hex.13975)

#
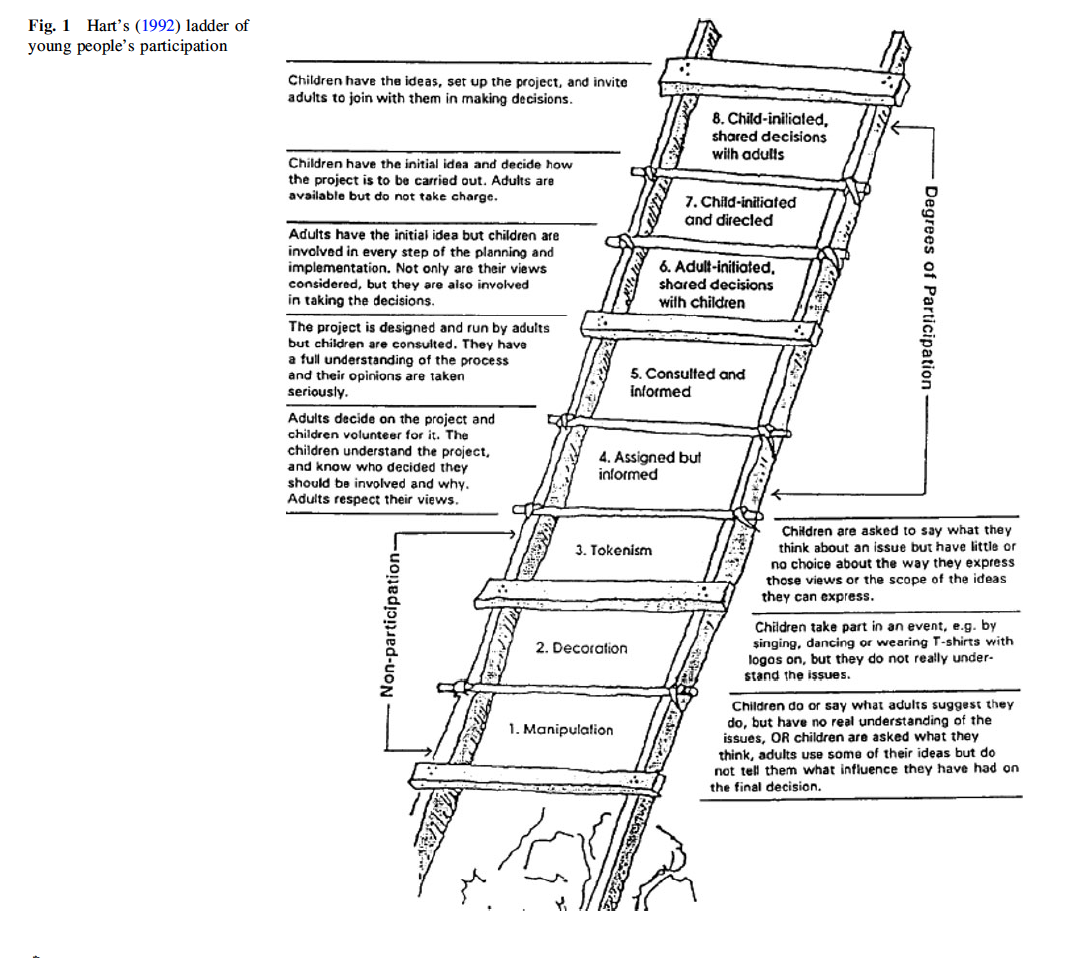
Power-focused frameworks (*n*=8)

**Power-focused framework 1**: Hart’s Ladder of Children’s Participation (1992)

**Power-focused framework 2**: Treseder’s Degrees of Participation (1997)


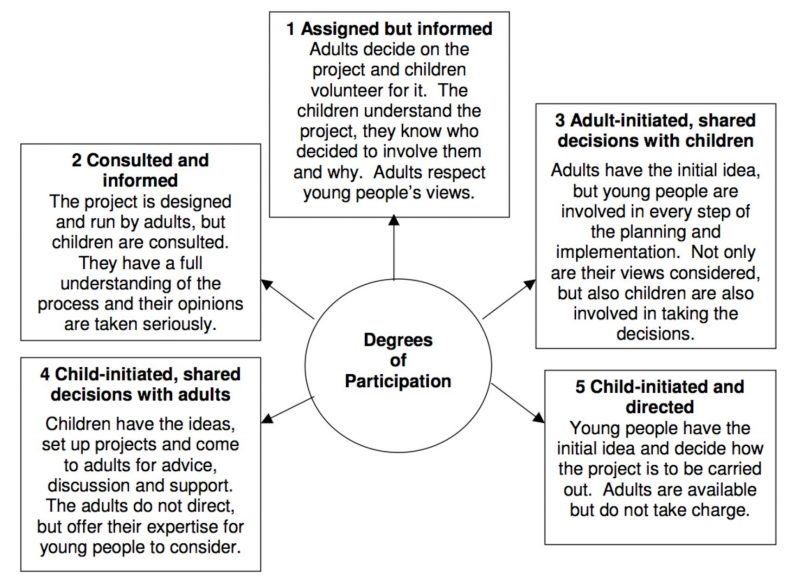


**Power-focused framework 3**: Shier’s Pathways to Participation (2001)


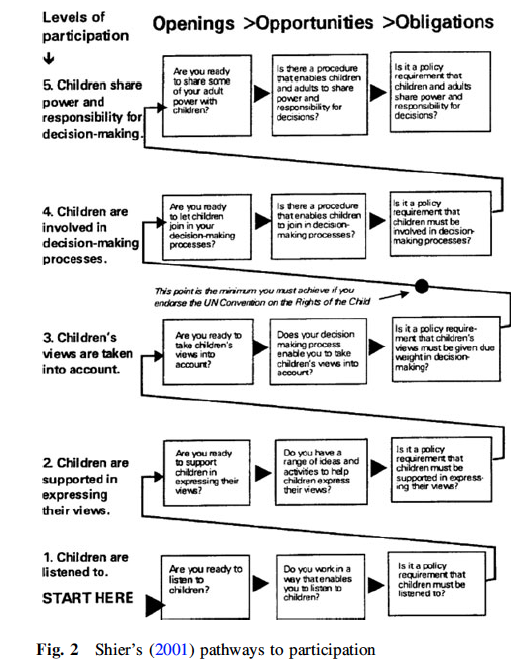


**Power-focused framework 4**: The TYPE Pyramid (2010)


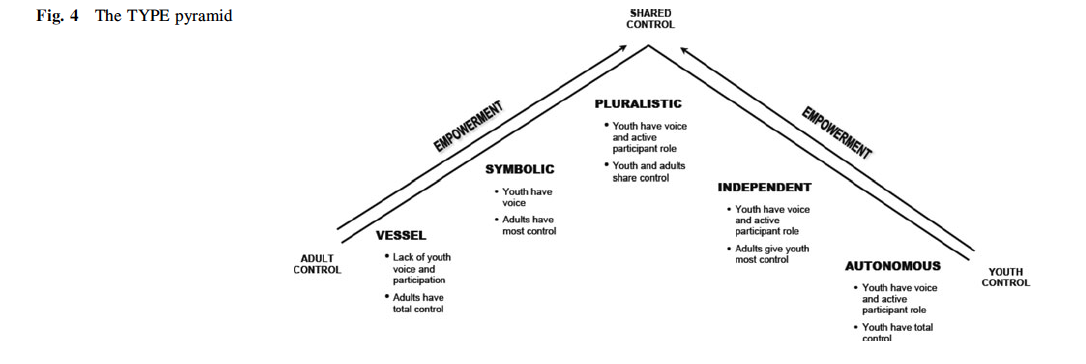


**Power-focused framework 5**: YPAR People Powered Place-Making (2016)


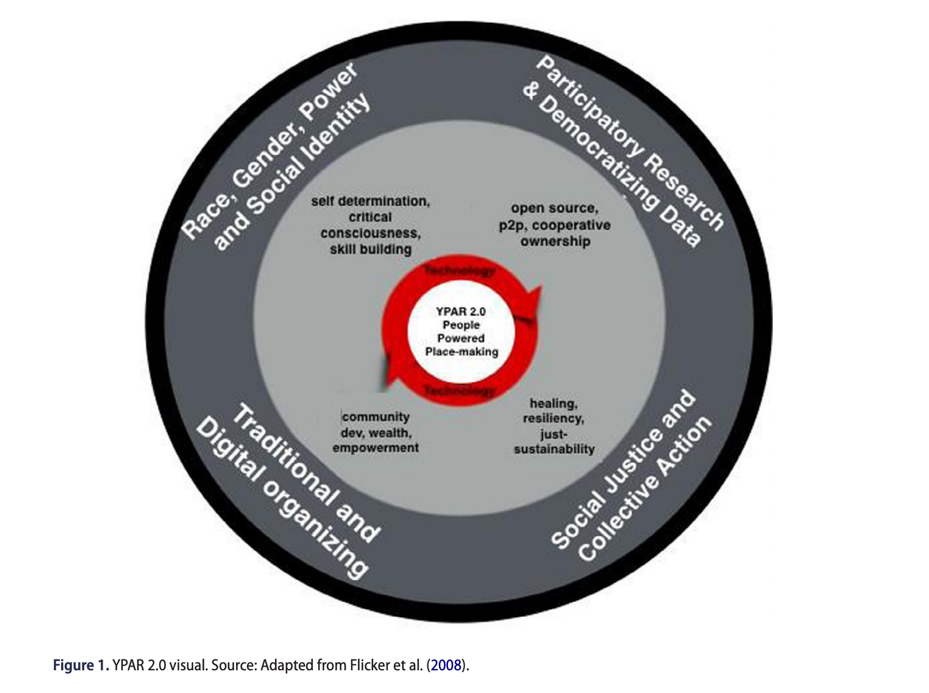


**Power-focused framework 6**: Ladder of Authentic Youth Participation (2018)


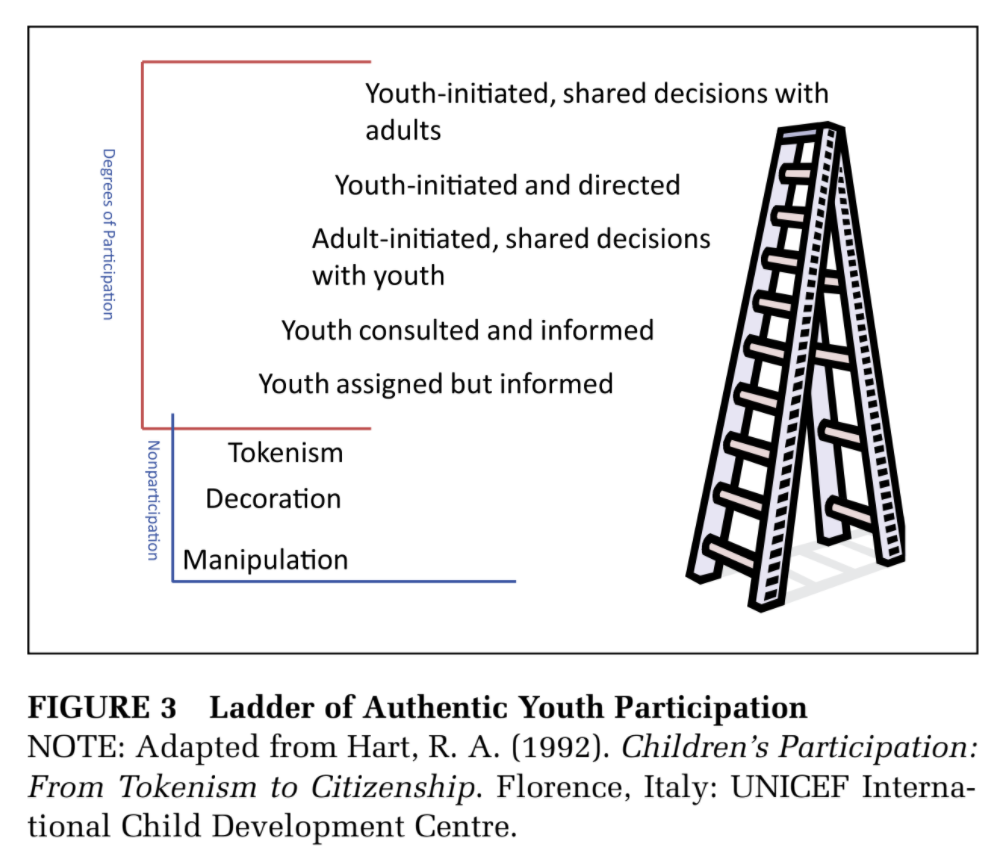


**Power-focused framework 7**: Levels of Youth Participation (2019)


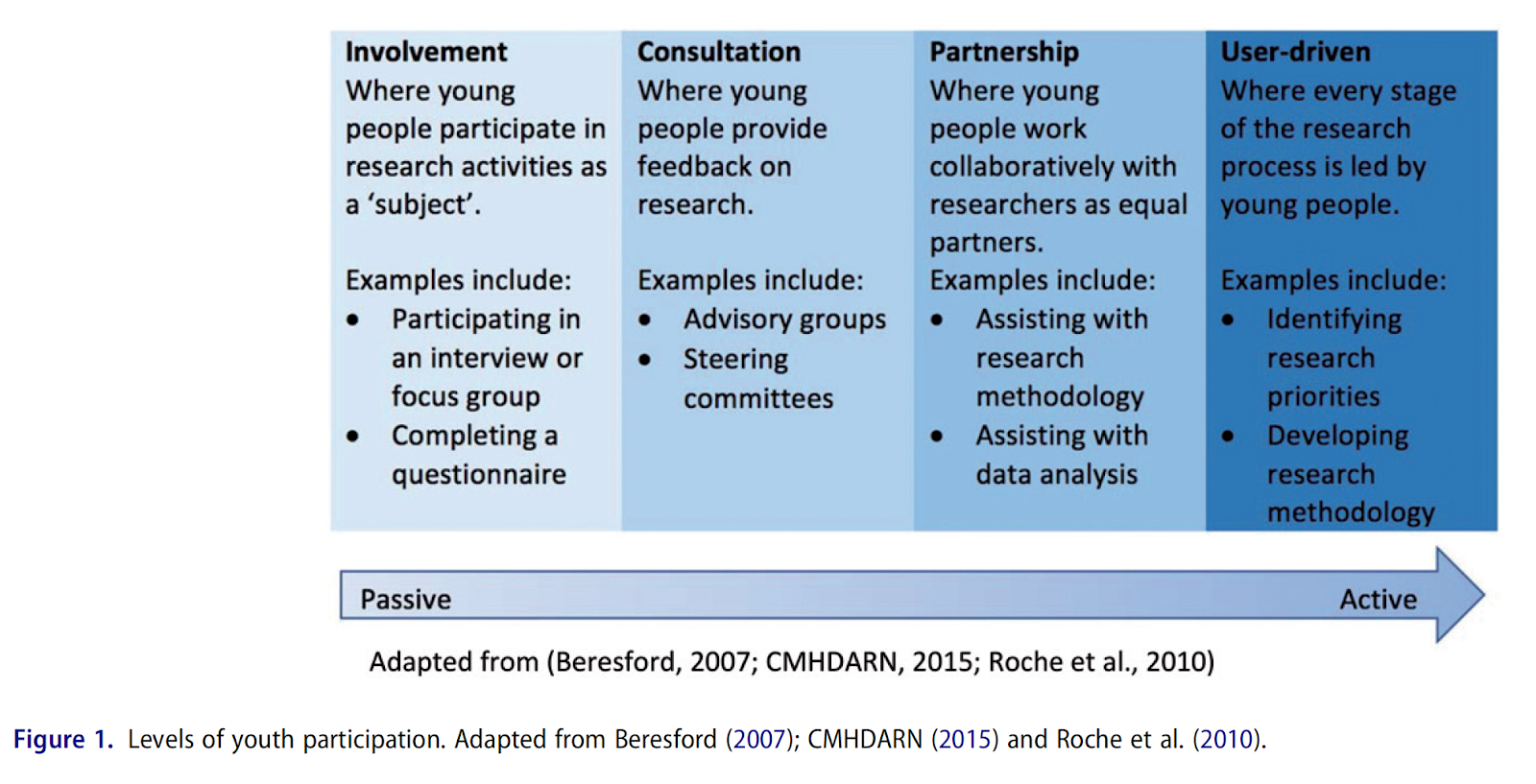


**Power-focused framework 8**: Measure of Youth Engagement (2021)


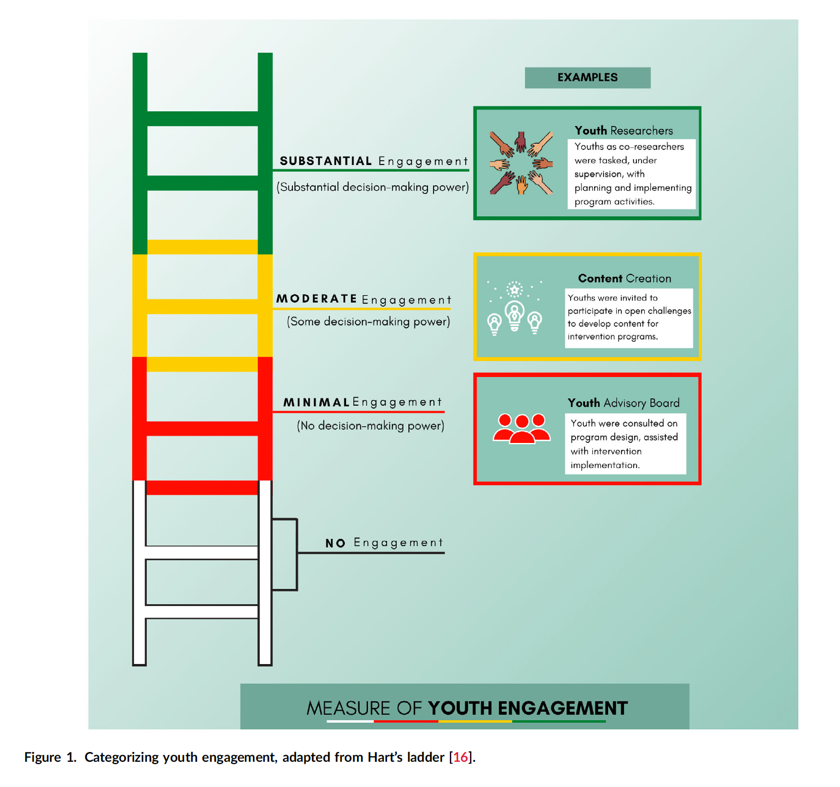


# Process-focused frameworks (*n*=7)

**Process-focused framework 1**: Youth Agency for Social Change Model (2006)


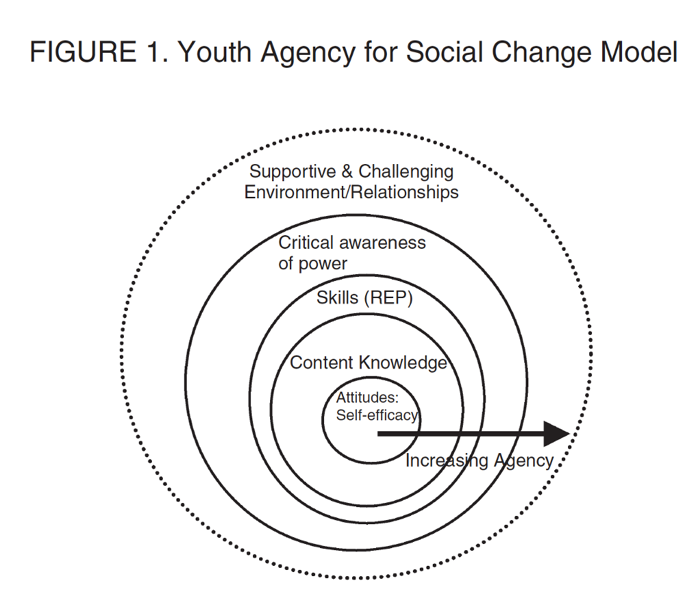


**Process-focused framework 2**: Model of Student Participation (2007)


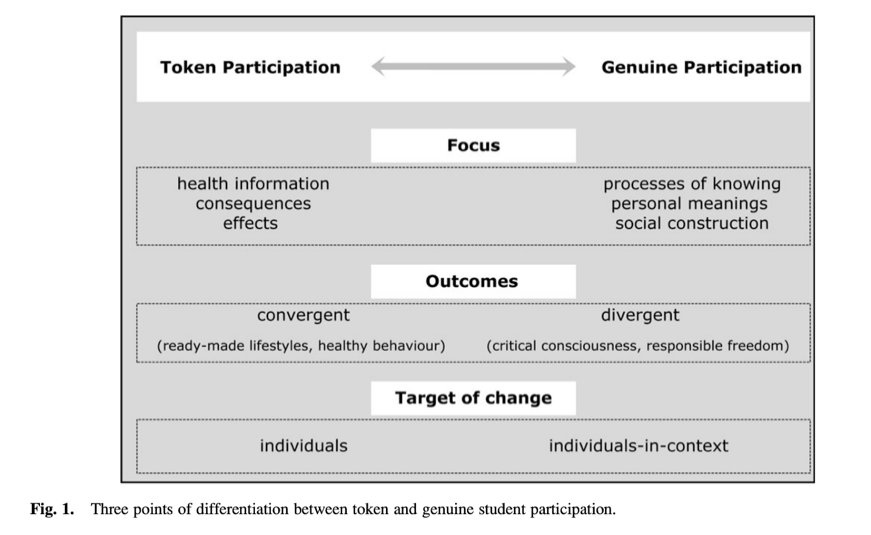


**Process-focused framework 3**: YPAR Continuum (2016)

**
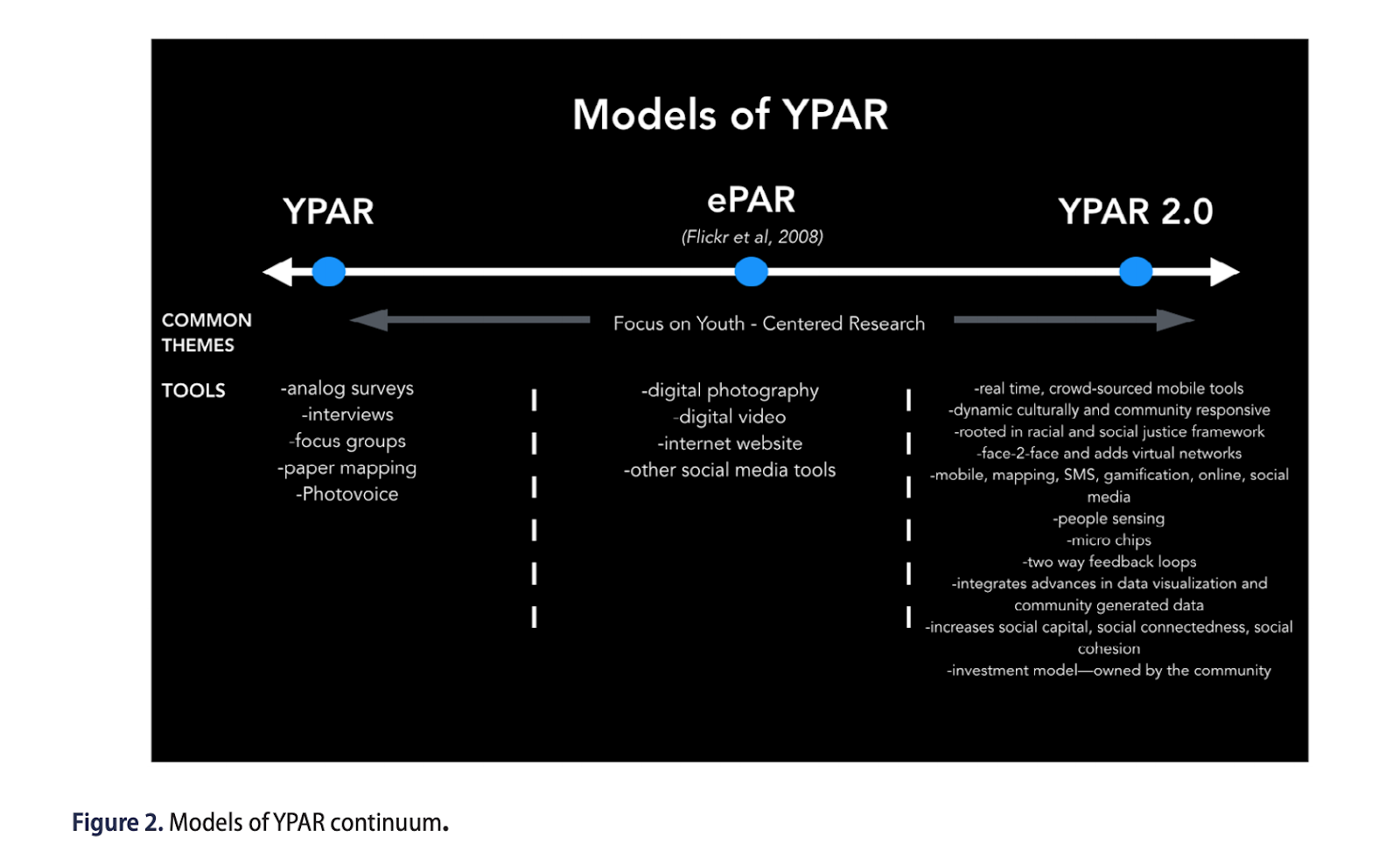
**

**Process-focused framework 4**: McCain Centre Model of Youth Engagement (2017)


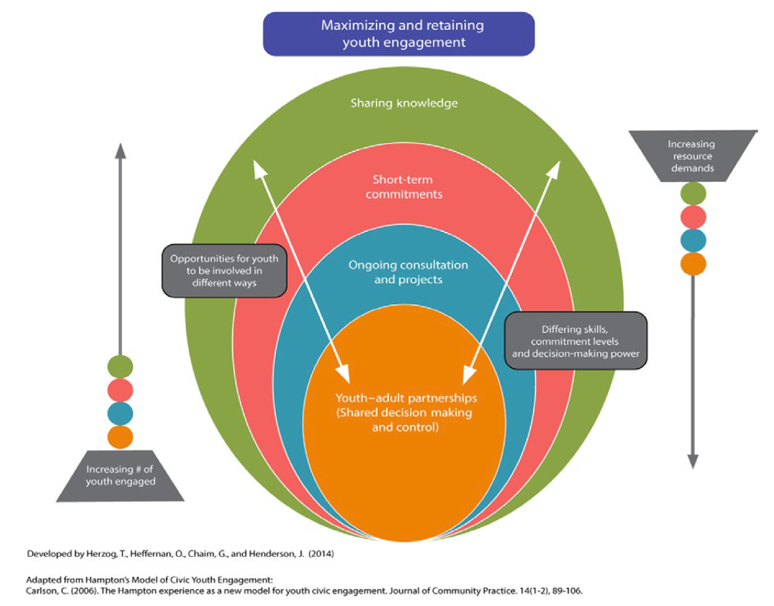


**Process-focused framework 5**: The P7 Model (2018)


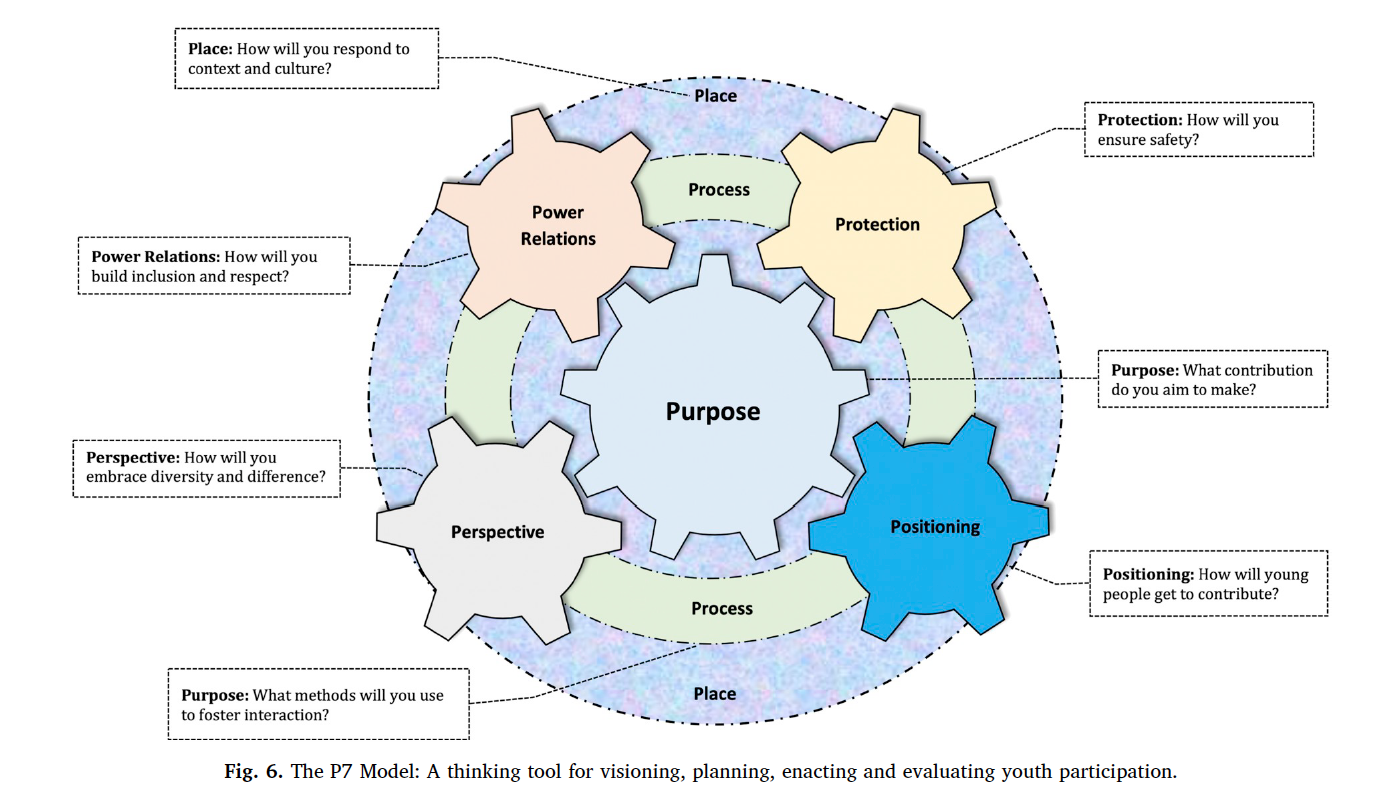


**Process-focused framework 6**: Youth Engagement for Community Change (2018)


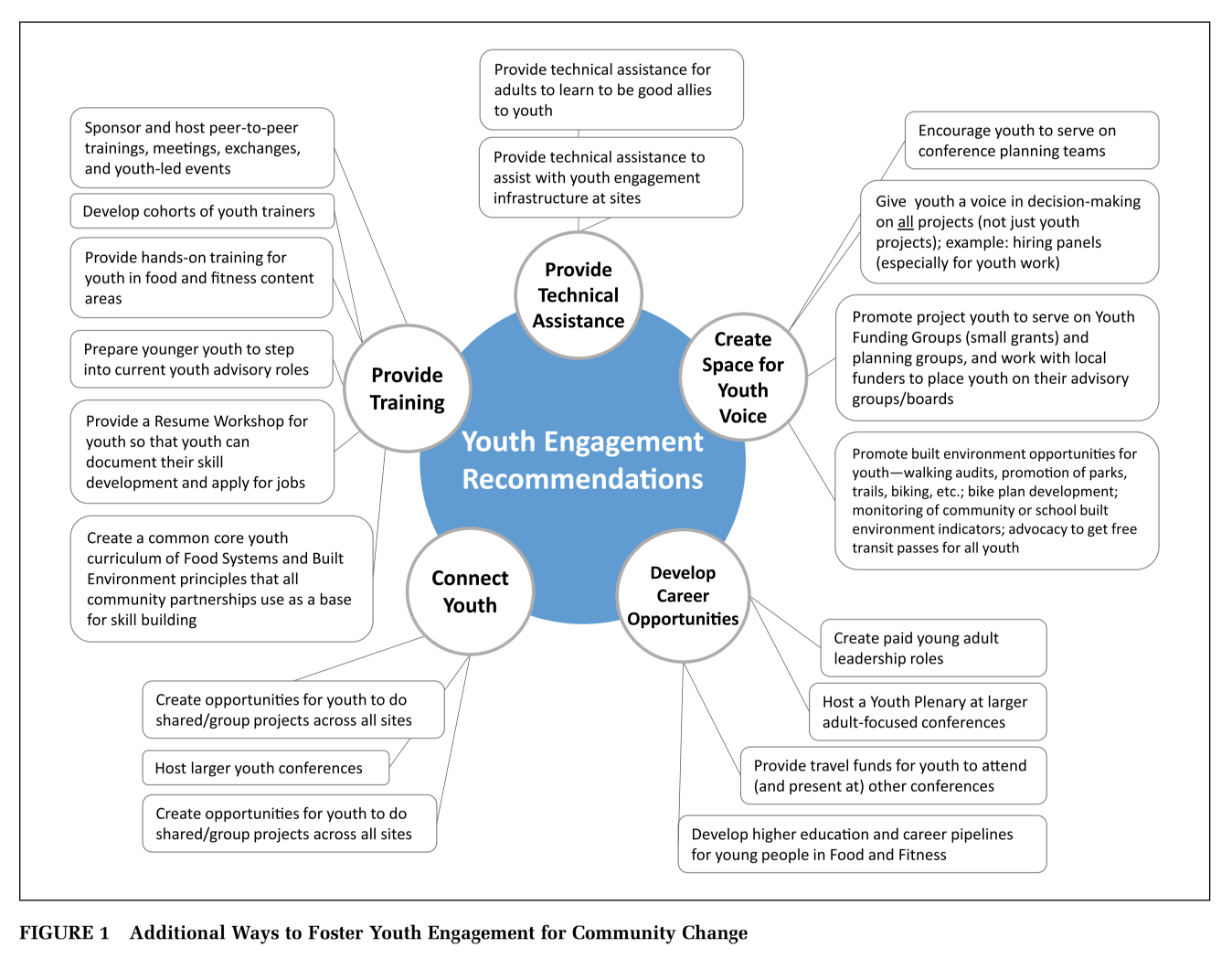


**Process-focused framework 7**: Making Youth Participation Genuine (2019)


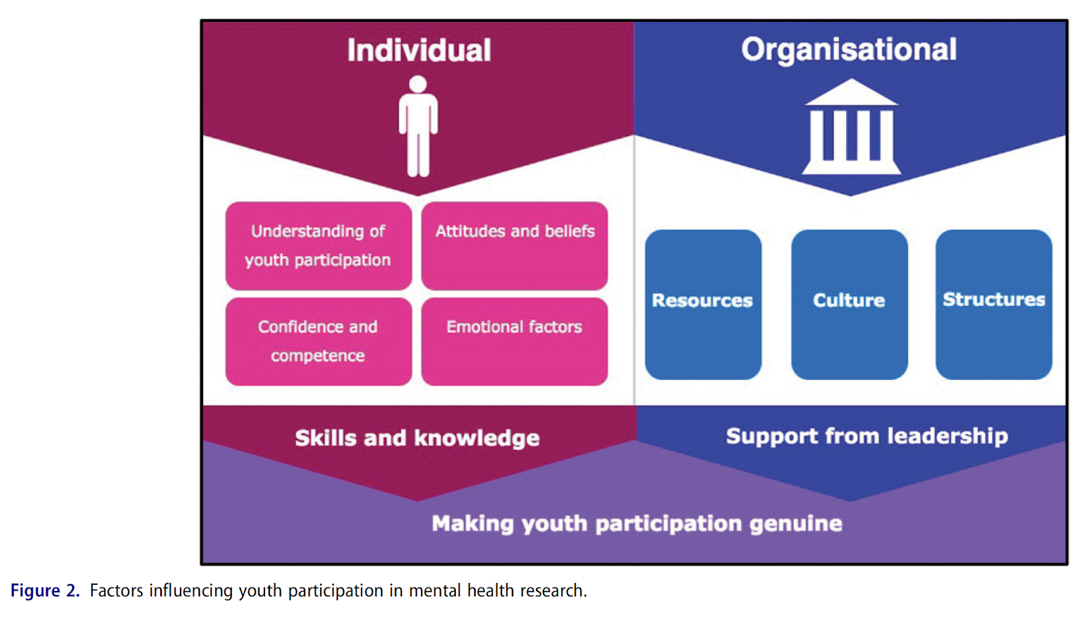


# Impact-focused frameworks (*n*=3)

**Impact-focused framework 1**: Youth Engagement Framework (2002)


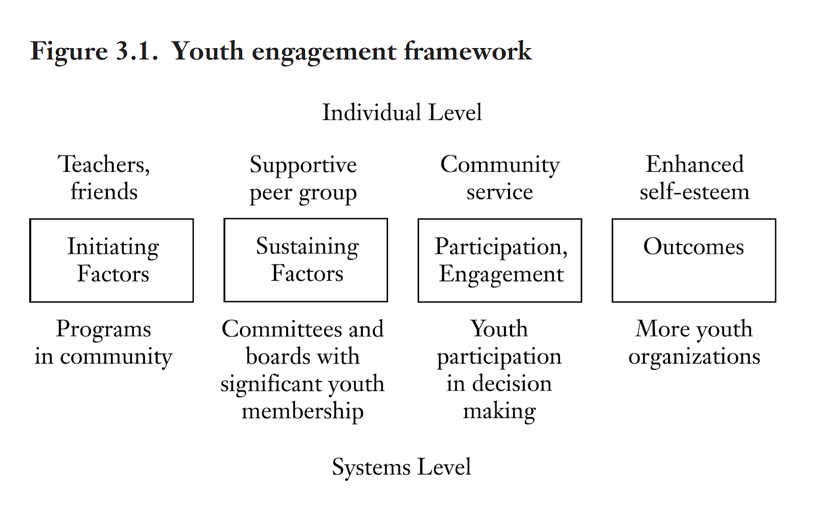


**Impact-focused framework 2**: EIPARS (2007)


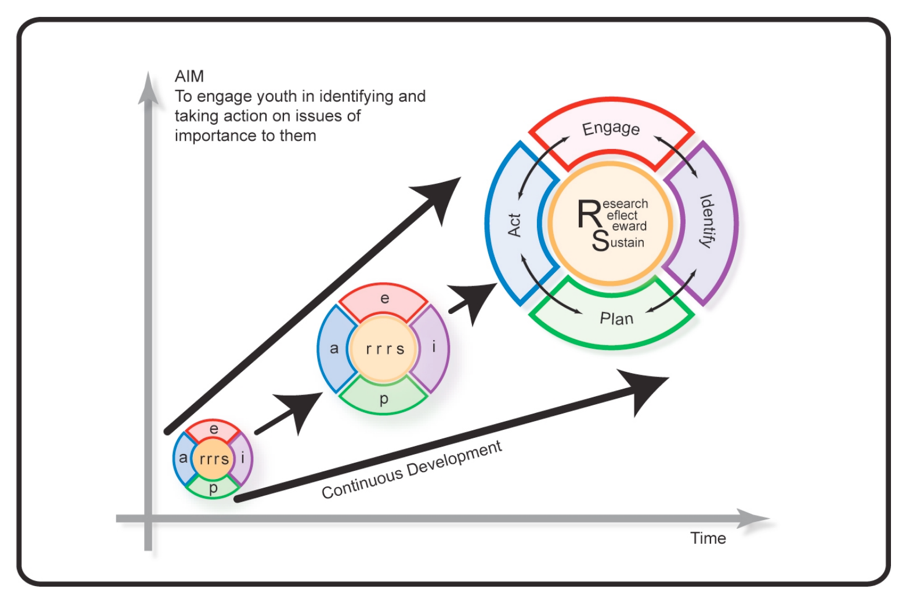


**Impact-focused framework 3**: Youth Engagement Framework by the Centre of Excellence for Youth Engagement (2009)


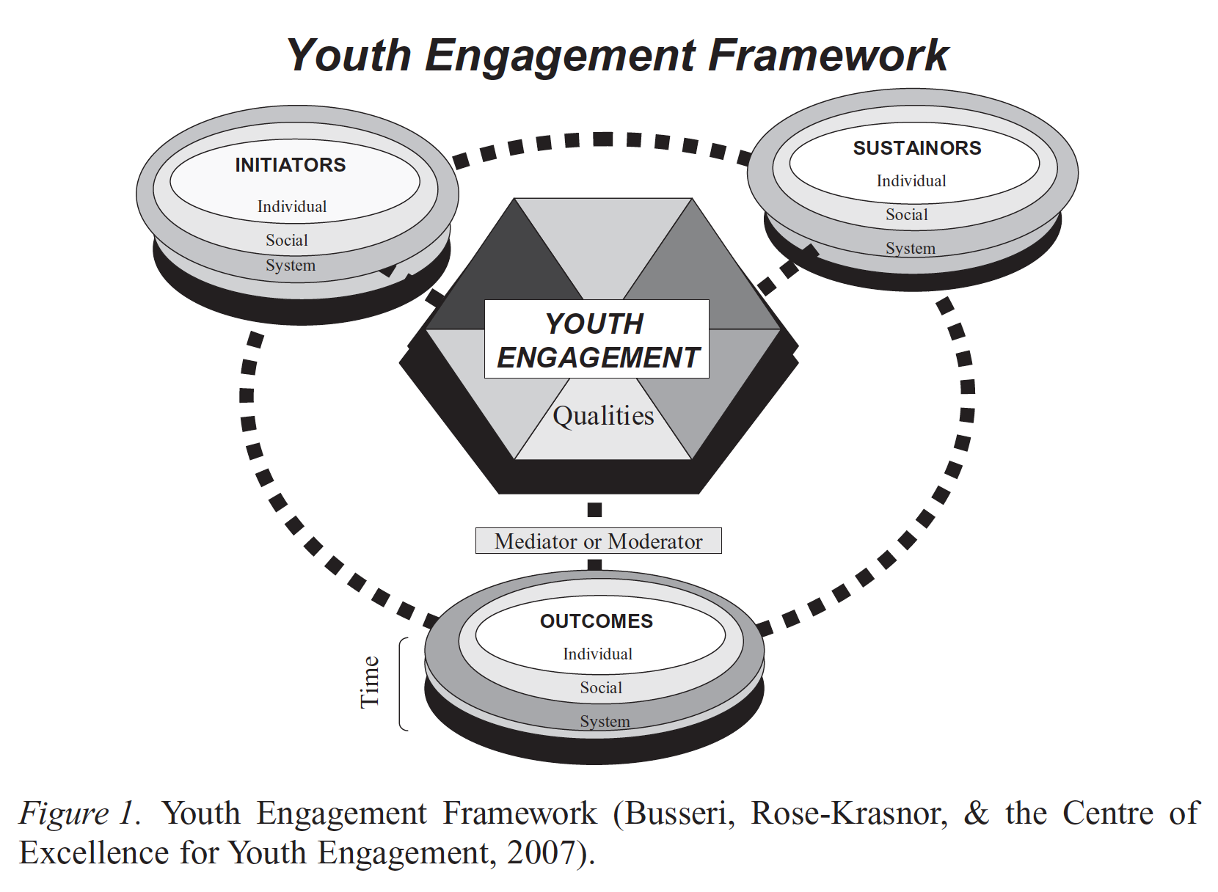


# Equity-focused frameworks (*n*=2)

**Equity-focused framework 1**: YPAR 2.0 Model of Research Engagement (2016)


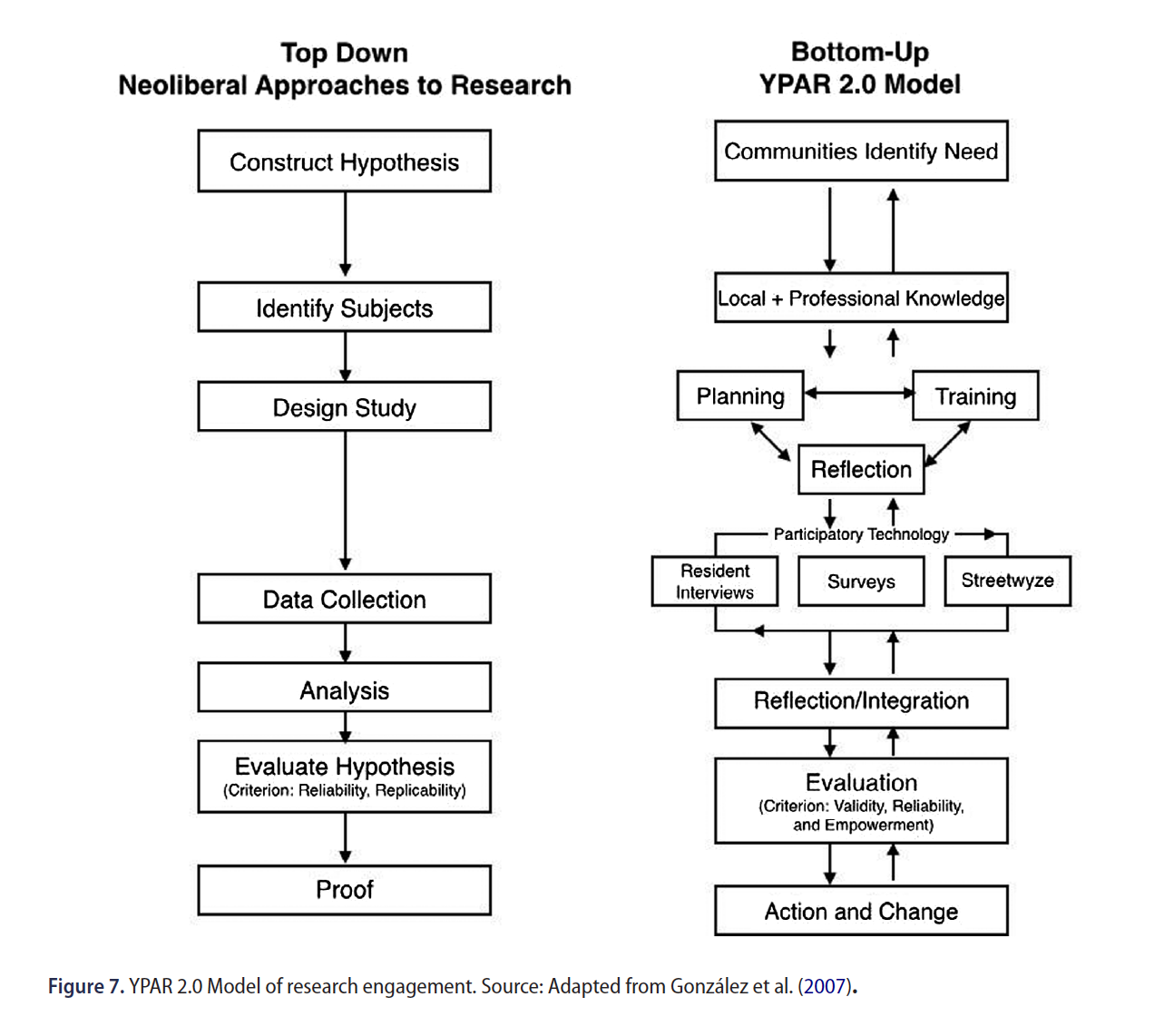


**Equity-focused framework 2**: Youth Participation Approaches Decision Tree (2020)


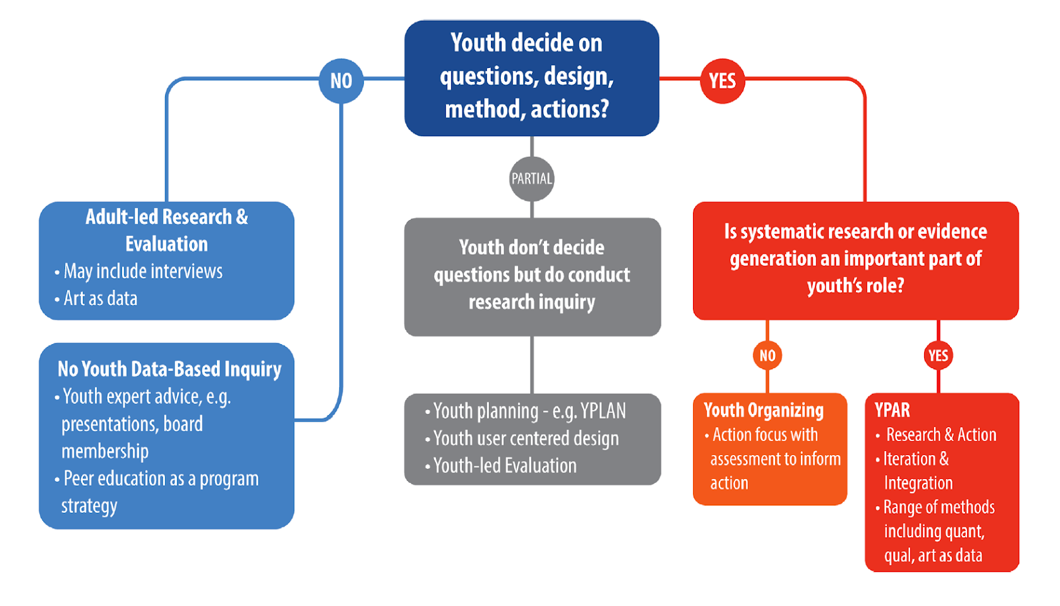

Supplement: Supplementary file 1 — Supporting information. [file HEX-27-e13975-s002.docx]
